# Brain Abscess Causes Brain Damage With Long-Lasting Focal Cerebral Hypoactivity that Correlates With Abscess Size: A Cross-Sectional ^18^F-Fluoro-Deoxyglucose Positron Emission Tomography Study

**DOI:** 10.1227/neu.0000000000003268

**Published:** 2024-11-11

**Authors:** Ebba Gløersen Müller, Daniel Dahlberg, Bjørnar Hassel, Mona-Elisabeth Revheim, James Patrick Connelly

**Affiliations:** * Division of Radiology and Nuclear Medicine, Department of Nuclear Medicine, Oslo University Hospital, Oslo, Norway;; ‡ Department of Neurosurgery, Oslo University Hospital, Oslo, Norway;; § Institute of Clinical Medicine, Faculty of Medicine, University of Oslo, Oslo, Norway;; ‖ Norwegian Defence Research Establishment (FFI), Kjeller, Norway;; ¶ Department of Neurohabilitation and Complex Neurology, Oslo University Hospital, Oslo, Norway;; # Division of Technology and Innovation, The Intervention Centre, Oslo University Hospital, Oslo, Norway

**Keywords:** Brain abscess, Brain activity, Brain metabolism, ^18^F-fluoro-deoxyglucose positron emission tomography, FDG-PET

## Abstract

**BACKGROUND AND OBJECTIVES:**

Bacterial brain abscesses may have long-term clinical consequences, eg, mental fatigue or epilepsy, but long-term structural consequences to the brain remain underexplored. We asked if brain abscesses damage brain activity long term, if the extent of such damage depends on the size of the abscess, and if the abscess capsule, which is often left in place during neurosurgery, remains a site of inflammation, which could explain long-lasting symptoms in patients with brain abscess.

**METHODS:**

2-[^18^F]-fluoro-2-deoxy-D-glucose positron emission tomography/computed tomography (FDG-PET/CT), electroencephalography, and MRI were performed 2 days to 9 years after neurosurgery for bacterial brain abscess.

**RESULTS:**

FDG-PET/CT revealed hypometabolism in the neocortex or cerebellum overlying the previous bacterial abscess in 38 of 40 patients. The larger the abscess, the greater was the extent of the subsequent hypometabolism (r = 0.63; p = 3 × 10^−5^). In 9 patients, the extent of subsequent hypometabolism seemed to coincide with the extent of peri-abscess edema in the acute phase. Follow-up MRI after ≥1 year in 9 patients showed focal tissue loss and gliosis. In 13 patients with abnormal electroencephalography recordings, abnormalities extended beyond the cerebral lobe affected by the abscess, indicating damage to wider brain networks. The abscess capsule had an FDG signal indicating inflammation only during the first week after neurosurgical pus drainage.

**CONCLUSION:**

The bigger a brain abscess is allowed to grow, the more extensive is the long-term focal reduction in brain activity. This finding emphasizes the need for rapid neurosurgical intervention. The abscess capsule does not display long-lasting inflammation and probably does not explain long-term symptoms after brain abscess.

## Graphical abstract

**Figure:**
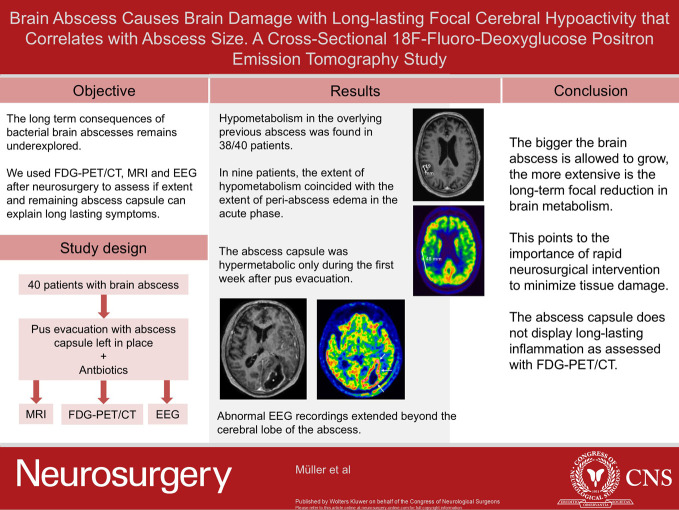

**ABBREVIATIONS:**
EEG: electroencephalography
FDG-PET: [^18^F]-fluoro-2-deoxy-D-glucose positron emission tomography.

A bacterial brain abscess results from a focal bacterial infection that leads to the formation of pus within the brain parenchyma.^1^ A fibrous capsule constitutes the abscess wall (Figure 1). A brain abscess could damage brain tissue in several ways. First, an abscess may contain many milliliters of pus that may cause tissue displacement and distortion. Second, the abscess pus contains many neuroactive and neurotoxic substances at high concentrations, such as bacterial toxins,^2,3^ cytokines,^4,5^ glutamate, potassium, and ammonia.^6-8^ It is at present unclear to what extent the abscess capsule prevents these molecules from diffusing into the surrounding brain tissue. Third, the abscess capsule is replete with leukocytes^1,9-11^ and constitutes a site of inflammation that could cause neurotoxicity. Long-term complications of brain abscess include epilepsy,^12^ cognitive disturbances,^13,14^ and mental fatigue^15^ support the notion that brain abscesses may cause permanent brain dysfunction or damage. We recently showed that brain abscesses expand day by day irrespective of efficient antibiotic therapy.^16^ This finding supported a recent guideline that a brain abscess should be drained of pus within 24 hours of diagnosis^17^; however, the guideline could be interpreted to mean that it is acceptable to delay neurosurgery by 24 hours from the time of diagnosis. A better understanding of long-term effects may inform the decision on when and how to treat patients with brain abscess.

**FIGURE 1.**
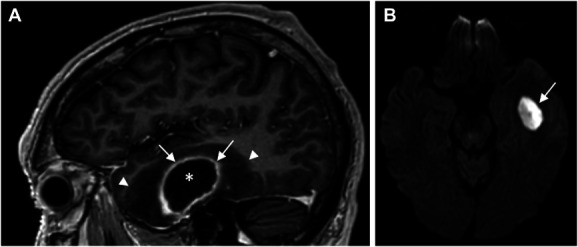
Brain abscess with capsule and surrounding edema. **A**, T1-weighted MRI after intravenous gadolinium contrast, showing an abscess in the left temporal lobe, with the abscess capsule (arrows) surrounding the abscess cavity (asterisk). Vasogenic edema surrounds the abscess (arrowheads). **B**, Diffusion-weighted MRI in the horizontal plane, showing highly restricted diffusion of the content of the abscess cavity (arrow), meaning that the content is highly viscous, ie, pus.

The purpose of this study was two-fold. First, knowing that brain abscesses expand with time,^16^ we wished to see if the degree of brain damage correlates with the size of the abscess. Second, we wished to see if the brain abscess capsule, if left in place during neurosurgery, represents a lasting focus of inflammation in the brain.

We used 2-[^18^F]-fluoro-2-deoxy-D-glucose positron emission tomography/computed tomography (FDG-PET/CT) to study brain activity in patients who had had neurosurgery for brain abscess from 2 days to 9 years previously. FDG accumulation corresponds closely to cellular glucose consumption, which in the brain is determined by neuronal activity.^18^ FDG-PET/CT may reveal brain damage that causes focal reduction of neuronal activity. This has been shown in patients with stroke, brain tumors, focal epilepsy, or multiple sclerosis.^19-22^ Furthermore, FDG-PET/CT may detect inflammation by showing the focal presence of leukocytes, which have a high demand for glucose.^23-25^ In the acute stage of brain abscess, the abscess capsule is rich in macrophages and microglia,^9-11^ and a high uptake of ^18^F-deoxyglucose in the abscess capsule has been documented.^26-32^ Less is known about the inflammatory state of the abscess capsule after surgical and antibiotic treatment. Furthermore, we examined patients with electroencephalography (EEG) and, when available, evaluated follow-up MRI results.

## METHODS

### Patients and Brain Abscess Treatment

Ethical approval was granted by The Regional Committees for Medical and Health Research Ethics of Norway (Approval # 1081/2018 and 256/2014). The study conformed to the Declaration of Helsinki^33^ and is registered at ClinicalTrials.gov (identifier: NCT04938362).

Fifty patients who had been treated for brain abscess at the Department of Neurosurgery, Oslo University Hospital, Oslo, Norway, January 2012 to July 2022, were consecutively invited to participate in the study (see **Supplemental Digital Content 1**, http://links.lww.com/NEU/E569 for flow chart). Seven patients declined the invitation. Spontaneously, 2 patients cited the distance to the hospital as too long, 2 felt the FDG-PET investigation would not benefit them, and 3 did not give a reason. Forty-three patients gave informed, written consent to participate. Three patients were excluded from the study. Two of these had brain abscess after meningioma extirpation, and one had a metal fragment in the brain; an abscess developed in the same area. We excluded these patients because their FDG-PET results could not be attributed solely to the brain abscess. Thus, 40 patients were included in the study.

In the acute stage of their brain abscess disease, patients underwent pus evacuation, either in a minimally invasive neurosurgical procedure that involved making a burr hole less than 10 mm in diameter in the cranium or in a more comprehensive procedure that involved making a craniotomy 30 to 40 mm in diameter. The abscess was punctured, and pus was drained through a steel cannula (diameter 2 mm) before the abscess cavity was rinsed once with saline. The abscess capsule was not removed in any of the patients. Antibiotics were given intravenously for at least 2 weeks and later perorally for at least 4 weeks. Six patients had to undergo pus evacuation twice because of continued infection. For microbial diagnosis, pus samples underwent aerobic and anaerobic culture and direct 16S ribosomal DNA polymerase chain reaction amplification and sequencing.

### MRI and FDG-PET/CT

Patients underwent 1.5 T MRI, which included T1-weighted imaging before and after intravenous infusion of a gadolinium contrast medium (Clariscan, GE Healthcare), T2-weighted imaging, T2-weighted fluid-attenuated inversion recovery imaging, diffusion-weighted imaging, and apparent diffusion coefficient mapping before neurosurgery. The volume of the abscess was calculated semiautomatically from postcontrast T1-weighted images with the Smartbrush program (Brainlab). In 3 cases, only a presurgery, contrast-enhanced CT scan was available.

For FDG-PET/CT, patients fasted for at least 6 hours, which gave a serum glucose value of 5.6 ± 1.0 mmol/L (mean ± SD). Patients were given FDG, 147 ± 30 MBq, intravenously, followed by PET after 64 ± 21 minutes with a scan time of 8.7 ± 2.0 minutes. PET images were acquired on a GE Discovery MI scanner (GE Healthcare). Scan duration was 8 minutes for adults older than 35 years and 10 minutes in those younger than 35 years, who received a lower dose of radioactivity. A low-dose CT scan was included for attenuation correction and anatomical information. The PET images were visually inspected with Siemens syngo.via (Siemens Healthineers, Erlangen, Germany) by 3 experienced nuclear medicine physicians/radiologists (M-ER, EGM, and JPC). Fourteen patients have been described previously regarding FDG-PET results.^16^

**FIGURE 2.**
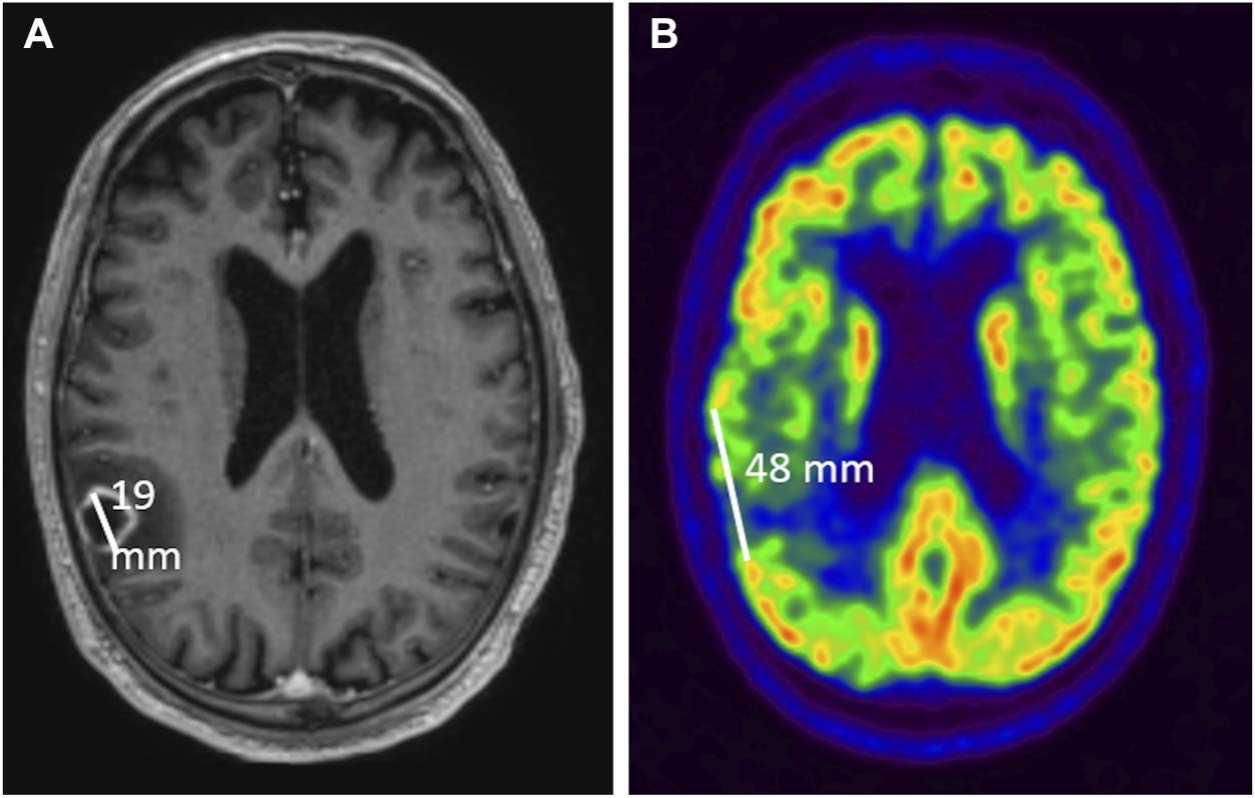
Measurement of abscess dimension and extent of neocortical hypometabolism. **A**, T1-weighted MRI shows the maximal diameter of the abscess in right parietal lobe in the horizontal plane. **B**, [^18^F]-fluoro-2-deoxy-D-glucose positron emission tomography/computed tomography in the same plane as image a, obtained 20 months later, shows the extent of neocortical hypometabolism in the area where the abscess was located. Note that the hypometabolic area exceeds the dimensions of the abscess.

To look for correlations between abscess size and extent of FDG-PET signal reduction, we measured the diameter of the abscess parallel to the overlying neocortex or cerebellar cortex in contrast-enhanced T1 MR images. In the same plane, we measured the extent of cortical tissue with reduced FDG signal in PET images (Figure 2).

### Electroencephalography (EEG)

EEG was recorded with 25 channels in the awake state for approximately 30 minutes. Recordings were conducted at various hospitals and with different equipment.

### Data Presentation and Statistics

FDG-PET data are given binarily as the presence or absence of focal hypometabolism. EEG findings are given as “normal,” “slow,” or “epileptiform” activity, and the distribution of EEG changes is described as the cerebral lobes affected. Correlations are given as Spearman coefficients. Group differences were analyzed with the Kruskal-Wallis test, Mann-Whitney *U* test, paired Student *t*-test, or the Fisher exact test, as appropriate. A *P*-value of <.05 was considered statistically significant. The data that support the findings of this study are available from the corresponding author on reasonable request.

## RESULTS

### Patient and Brain Abscess Characteristics

Patient and abscess characteristics are given in Table 1. Of the 40 patients, 38 had a single brain abscess with a median volume of 7.0 cm^3^ (range 0.6-42). Two patients had multiple abscesses that were located in various cerebral lobes and in the cerebellum. One of these, who had brain abscess due to endocarditis, had 9 abscesses ranging from <0.1 to 3.1 cm^3^, another patient had 5 abscesses ranging from 1.0 to 3.9 cm^3^. Abscess size did not differ significantly regarding the location of the abscess (*P* = .61).

**TABLE 1.** Patient and Abscess Characteristics

Women (n = 13) age (y, median, full range)	55 (30-71)
Men (n = 27) age (y, median, full range)	60.6 (21-79)
Abscess volumes cm^3^ (median value, full range)	
Frontal abscesses (n = 13)	9.9 (1.4-26)
Temporal abscesses (n = 9)	4.8 (0.6-42)
Parietal abscesses (n = 7)	3.3 (1.7-18)
Occipital abscesses (n = 6)	11.0 (0.6-39)
Cerebellar abscesses (n = 2)	6.5 and 16
Basal ganglia abscess (n = 1)	7.2

Of 40 patients with brain abscess, 38 had a solitary abscess. The table gives their lobar location and volumes (cm^3^).

Four patients with a frontal abscess had contralateral motor symptoms ranging from mildly reduced speed to severe hemiparesis. Two patients with a frontal lobe abscess had motor dysphasia. Two patients with an occipital abscess had homonymous hemianopsia. Eleven patients developed focal epilepsy after abscess neurosurgery (see: *EEG findings and epilepsy*). The rest of the patients did not have focal neurological symptoms, but 20 of the 40 patients complained of mental fatigue that had developed after the brain abscess.

In all but 2 patients, a microbial diagnosis was achieved. In 14 pus samples, several bacterial species were identified. *Streptococcus intermedius* was identified in 27 pus samples, *Fusobacterium* species (*nucleatum, necrophorum*) in 9, and *Aggregatibacter aphrophilus* in 7. Other isolates were *Streptococcus constellatus, Streptococcus gordoni*, *Streptococcus pneumoniae*, *Actinomyces meyeri, Anaeroglobus germinatus*, *Campylobacter rectus*, *Citrobacter* species, *Eubacterium brachy*, *Gemella morbillorum*, *Haemophilus parainfluenzae*, *Parvimonas micra*, *Porphyromonas endodontalis*, and *Prevotella oris*.

### MRI and FDG-PET/CT Findings

**FIGURE 3.**
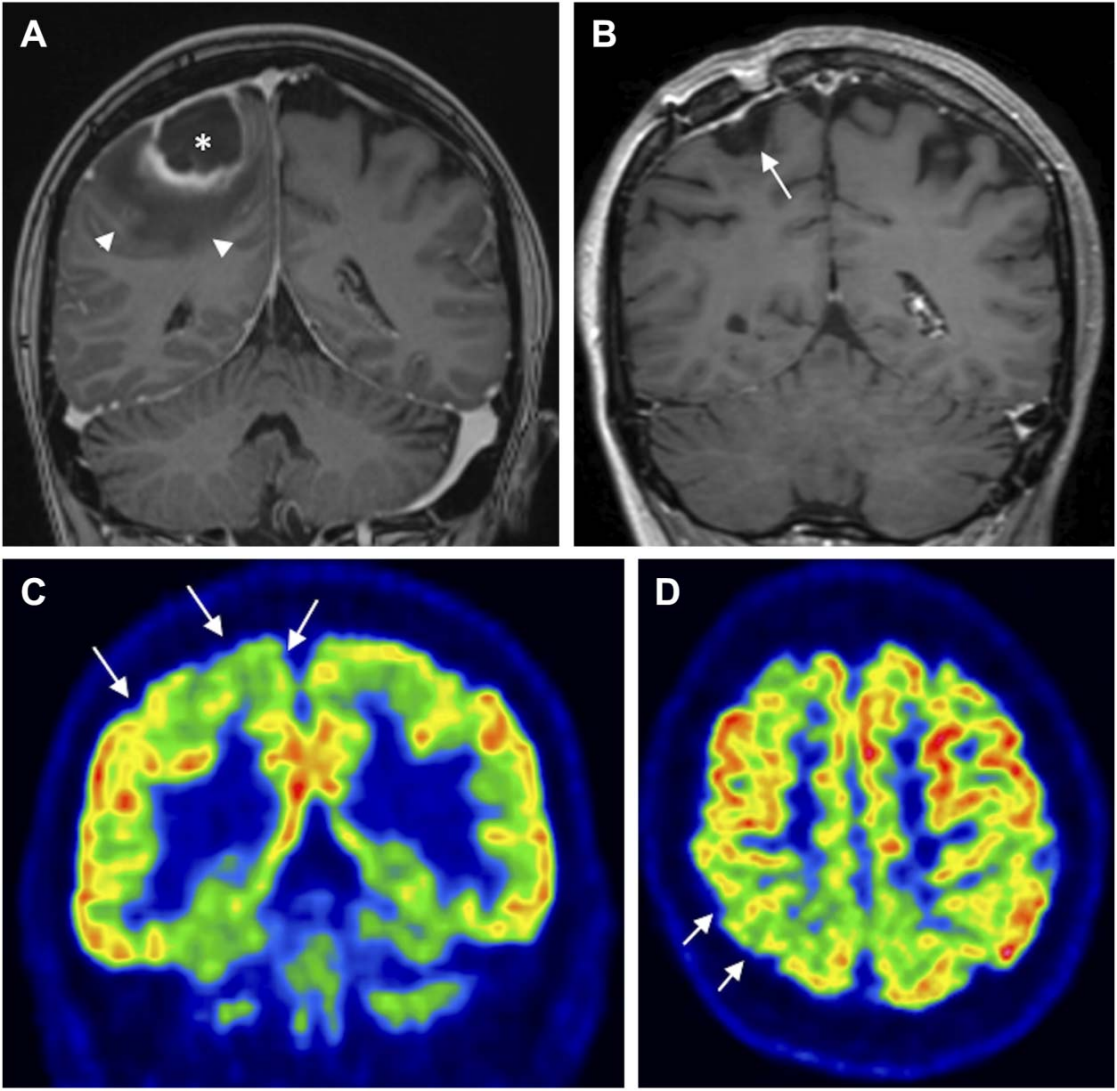
Tissue loss and hypometabolism after brain abscess. **A**, MRI showing an abscess (*) in the right parietal lobe surrounded by vasogenic edema (arrowheads) before pus removal; **B**, cortical tissue loss 1 year later (arrow). **C** and **D**, [^18^F]-fluoro-2-deoxy-D-glucose positron emission tomography/computed tomography (coronal and axial sections, respectively) in the same patient 3 years after pus removal, showing neocortical hypometabolism (arrows).

T1-weighted MRI of the brain before neurosurgery showed a cavity bounded by gadolinium contrast enhancement and vasogenic edema in the surrounding brain tissue (Figures 1A and 3A). Diffusion-weighted MRI showed highly restricted diffusion of the content of the cavity, indicating pus (Figure 1B).

**FIGURE 4.**
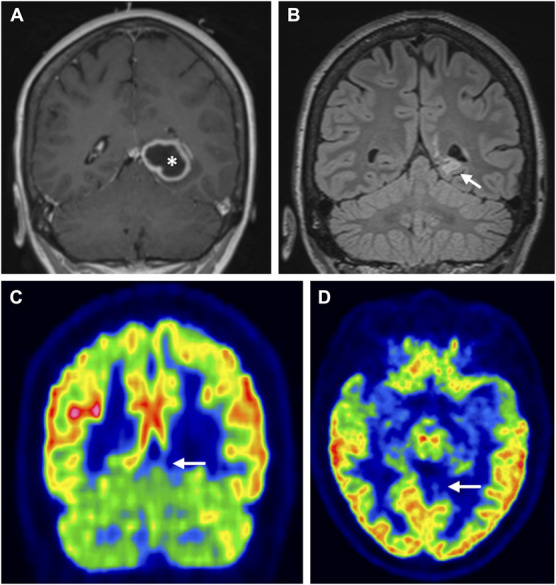
Gliosis and hypometabolism after brain abscess. **A**, T1-weighted postcontrast MRI showing an abscess (*) in the left precuneus before pus removal; **B**, T2-weighted fluid-attenuated inversion recovery MRI showing gliosis in the area of the abscess 2 years after pus removal (arrow). **C** and **D**, [^18^F]-fluoro-2-deoxy-D-glucose positron emission tomography/computed tomography (coronal and axial sections, respectively) in the same patient, showing neocortical hypometabolism (arrows) 2 years after pus removal.

For 9 patients, MRIs obtained a year or more (median 1.75 years; range 1-5) after neurosurgery were available for the evaluation of long-term structural effects of the abscesses. In all 9 patients, a variable degree of tissue loss (Figure 3) and subcortical gliosis (Figure 4) could be seen in the area of the previous abscess (see **Supplemental Digital Content 2**, http://links.lww.com/NEU/E570 for all 9 patients).

**FIGURE 5.**
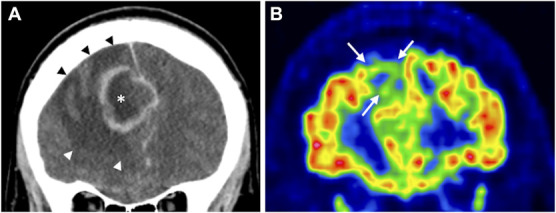
Neocortical hypometabolism 9 years after brain abscess. **A**, Coronal CT scan showing an abscess (*) in the right frontal lobe causing midline shift before pus removal. Note peri-abscess vasogenic edema (black and white arrowheads). **B**, [^18^F]-fluoro-2-deoxy-D-glucose positron emission tomography/CT (coronal section) of the same patient 9 years after pus removal, showing widespread neocortical hypometabolism in the area of the previous abscess (arrows). CT, computed tomography.

**FIGURE 6.**
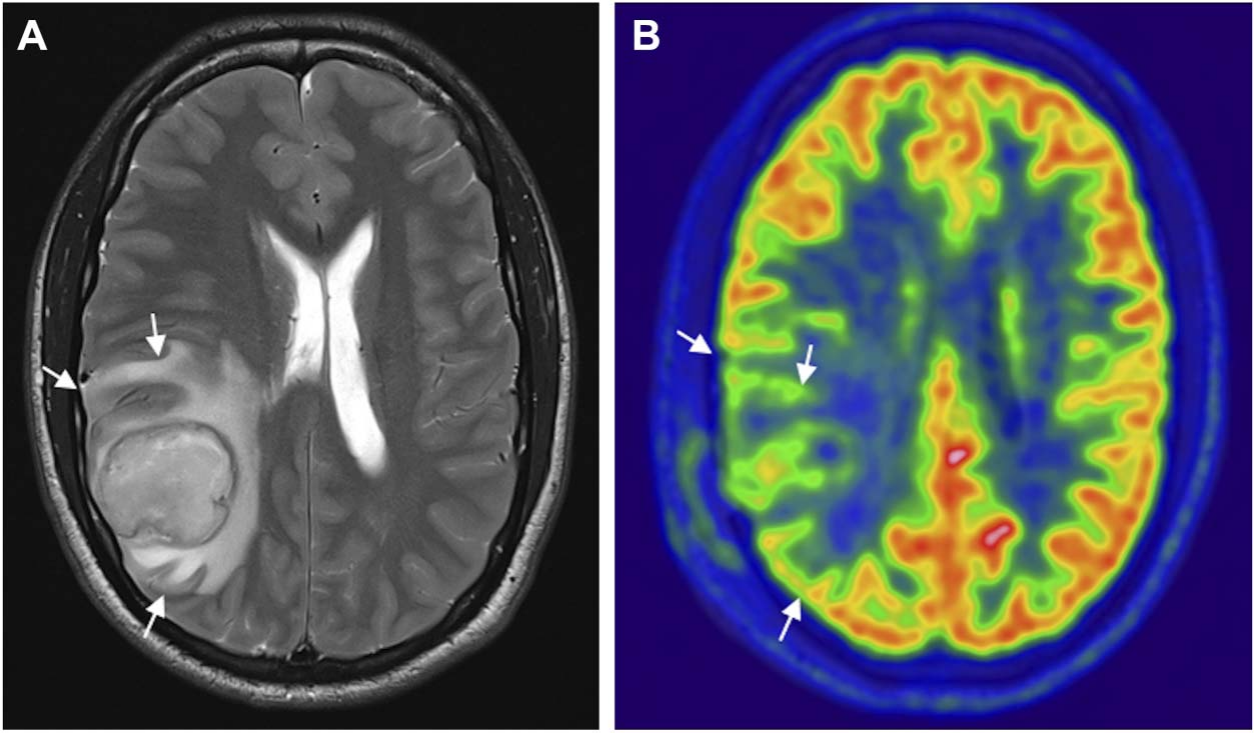
Hypometabolism corresponding to previous peri-abscess vasogenic edema. **A**, Axial T2-weighted MRI showing an abscess (*) in the right parietal lobe before pus removal. Vasogenic edema surrounds the abscess (arrows). **B**, Axial [^18^F]-fluoro-2-deoxy-D-glucose positron emission tomography 2 years after pus removal shows neocortical hypometabolism that corresponds to the area previously occupied by the vasogenic edema (arrows).

All 40 patients underwent FDG-PET/CT 2 days to 9 years after neurosurgery for brain abscess (median: 630 days). In all but 2 cases, there was marked reduction in FDG uptake in the neocortex or cerebellar hemisphere overlying the previous abscess, indicating reduced neuronal glucose metabolism and reduced neuronal activity (Figures 2-5). There was a rather distinct border between the hypometabolic neocortical or cerebellar areas and the adjacent brain tissue with normal FDG uptake. The FDG signal varied somewhat within the hypometabolic cortical areas, but at its lowest, it was similar to that of white matter (Figures 2-6). The 2 patients who did not display detectable neocortical reduction in FDG uptake had had small abscesses deep within the forebrain white matter. In some patients who harbored multiple abscesses, no focal reduction in FDG uptake could be seen corresponding to the smallest abscesses (<0.5 cm^3^).

**FIGURE 7.**
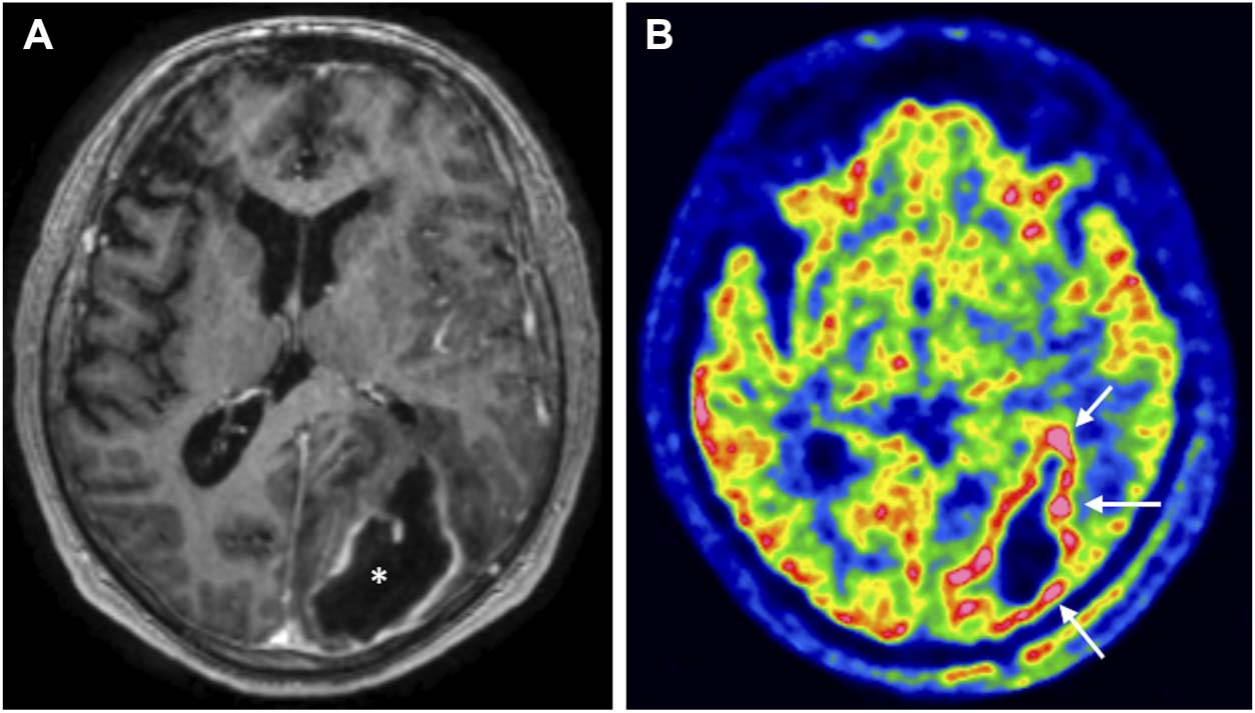
FDG uptake in the abscess capsule. **A**, A patient with a large abscess in the left occipital lobe (asterisk; T1-weighted MRI). **B**, [^18^F]-fluoro-2-deoxy-D-glucose positron emission tomography/computed tomography obtained 2 days after pus removal. There is FDG uptake corresponding to the brain abscess capsule (arrows). This finding implies the presence of leukocytes in the capsule that convert FDG to FDG-6-phosphate. FDG, [^18^F]-fluoro-2-deoxy-D-glucose.

In 9 patients who underwent FDG-PET/CT years after brain abscess treatment, the extent of neocortical hypometabolism seemed to coincide with the area that had been edematous at the time of the abscess (Figure 6; see **Supplemental Digital Content 3**, http://links.lww.com/NEU/E571 for all 9 patients). In 2 patients who underwent FDG-PET/CT 2 and 6 days after brain abscess surgery, respectively, FDG uptake could be seen corresponding to the abscess capsule (Figure 7). In the remaining patients, who underwent FDG-PET/CT 21 days after surgery, or later, no FDG signal corresponding to the abscess capsule could be seen.

**FIGURE 8.**
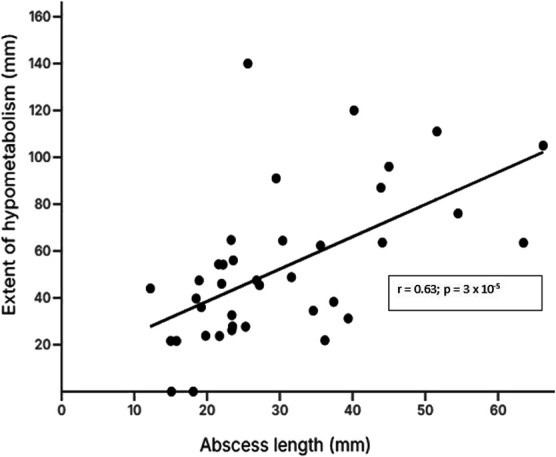
Extent of hypometabolism vs abscess dimension. Forty patients with brain abscess underwent preoperative MRI and FDG-PET/CT 2 days to 9 years after neurosurgery. The figure shows the correlation between the maximal length of the abscess before neurosurgery as determined from preoperative MRI and, in the same plane, the maximal extent of cortical (neocortical or cerebellar) hypometabolism as determined from FDG-PET/CT images. The correlation was determined by the Spearman test. FDG-PET/CT, [^18^F]-fluoro-2-deoxy-D-glucose-positron emission tomography/computed tomography.

We measured the maximal diameter of the abscess parallel to the overlying neocortex or the cerebellar cortex and measured the length of the overlying cortex with reduced FDG uptake (Table 2; Figure 2). In 38 patients, focal hypometabolism was evident. In 36 of these 38, the length of hypometabolic neocortex or cerebellar cortex exceeded the dimension of the brain abscess; in the remaining 2 patients, abscess dimension and extent of hypometabolism were the same. Moreover, the extent of neocortical or cerebellar hypometabolism correlated with the dimension of the abscess (Table 2; Figure 8), meaning that the larger the abscess, the greater the extent of subsequent neocortical or cerebellar hypometabolism. Looking only at the 27 patients who underwent FDG-PET/CT more than 1 year after neurosurgery (median value 36.3 months; range 15-104), the extent of focal hypometabolism still exceeded the dimension of the abscess: 47 mm (median value; range 0-140) vs 26 mm (range 15-55; *P* = .00032; Student 2-tailed paired *t*-test), and the extent of focal hypometabolism correlated with the dimension of the abscess: r = 0.60 (*P* = .0017; Spearman test), showing that the extensive focal hypometabolism was a persistent effect.

**TABLE 2.** Abscess Dimension and Extent of Focal Hypometabolism After Brain Abscess

Abscess diameter by MRI (median value; range; n = 40)	25 mm (12-66)
Length of cortical hypometabolism by FDG-PET (median value; range; n = 40)	46 mm (0-140)^a^
Correlation abscess diameter—extent of hypometabolism (n = 40)	0.63 (*P* = 3 × 10^−5^)

FDG-PET, [^18^F]-fluoro-2-deoxy-D-glucose positron emission tomography.

Forty patients who had had a brain abscess in any of the cerebral lobes or in the cerebellum underwent MRI before neurosurgery and FDG-PET 2 days – 9 years after neurosurgery. The abscess dimensions were measured from MRIs and are given (in mm) as the greatest diameter parallel to the overlying neocortex or cerebellar cortex. The extent of hypometabolism was measured from FDG-PET images and is given (in mm) as the length of hypometabolism in the neocortex or cerebellar cortex overlying the previous abscess. All but 2 patients who had small abscesses deep within the forebrain white matter displayed focal hypometabolism.

a Extent of hypometabolism different from abscess diameter; *P* = 9 × 10^−6^ (Student paired, two-tailed *t*-test). The correlation between abscess diameter and extent of hypometabolism was calculated with the Spearman test.

### EEG Findings and Epilepsy

All 40 patients underwent EEG at some time point between 2 days and 7 years (median 255 days) after brain abscess surgery. Fifteen patients with forebrain abscesses had abnormal EEGs. In all of these, slow activity (theta or delta activity) was seen over the area affected by the brain abscess. Two patients underwent EEG 2 days after neurosurgery; 1 of these had diffuse slow activity, and the other had normal EEG results.

In 13 of the 15 patients with abnormal EEGs, the abnormalities extended beyond the lobe affected by the abscess, indicating dysfunction of neuronal networks extending outside the affected lobe. The 2 patients with a single cerebellar abscess had normal EEG recordings. Eleven patients developed epilepsy after abscess neurosurgery.

## DISCUSSION

### Brain Abscess Causes Long-Lasting Focal Reduction in Neuronal Activity

We show here that brain abscess leads to focal reduction in neocortical or cerebellar metabolic activity, as detected with FDG-PET/CT. Reduced cerebral glucose metabolism is due to a reduction in neuronal activity^18^ and indicates brain damage. In many patients, the reduced metabolic activity was evident several years after the brain infection, and so, it was likely permanent. This finding points to the need for rapid neurosurgery in patients with brain abscess to minimize brain damage. This is all the more important because it has been shown that brain abscesses increase in size from one day to the next.^16^ We show in this study that the larger the abscess, the greater is the extent of cerebral hypometabolism, which means that the longer the abscess is allowed to expand, the greater will the damage to the brain be. Recent guidelines recommend neurosurgical drainage of a brain abscess within 24 hours of diagnosis.^17^ We would argue that the sooner the abscess is drained of pus, the greater will be the benefit to the patient.

The reduction in cortical FDG uptake (ie, glucose metabolism) after brain abscess may have several explanations. The focal loss of brain tissue coinciding with the site of the previous abscess would clearly lead to reduction in FDG signal. Subcortical gliosis implies damage to afferent axons in the subcortical white matter, which could reduce cortical activation, as has been reported in other brain disorders.^19-22^ In the days or weeks after neurosurgery, the vasogenic edema surrounding the abscess remnants would probably contribute to the reduction in metabolic activity, as has been seen in peri-tumoral or postirradiation vasogenic edema.^34-36^ Interestingly, in some patients who underwent FDG-PET/CT years after abscess surgery, the area of neocortical hypometabolism corresponded to the area that had been edematous in the acute stage of the disease rather than to the area of the abscess, suggesting a toxic effect of the edema itself, eg, of plasma proteins.^37^ Last, patients who developed an epileptic focus in the area of the abscess could have had cortical hypometabolism somehow related to their epileptic activity.^21-38^

The FDG signal in the hypometabolic areas varied somewhat, but at its lowest, it was similar to that of white matter. Glucose metabolism in human white matter is 50% to 75% of that in neocortex.^39,40^ Thus, a rough estimate would be that the metabolic activity within the hypometabolic area could be reduced by as much as 25% to 50%. We cannot say if this corresponds to a similar reduction in neuronal activity because both neurons and glial cells contribute importantly to cerebral glucose metabolism and thus to the FDG signal.^41,42^ Therefore, the focal reduction in neuronal activity after brain abscess could be both greater and smaller than the 25% to 50% estimate.

FDG uptake into the abscess capsule was only seen during the first week after pus drainage (in 2 patients), which is in line with a previous observation.^43^ This finding suggests that the abscess capsule does not remain a long-term focus of high-grade inflammation with a high influx of leukocytes; these would have been visualized with FDG-PET due to their high demand for glucose.^23-25^ However, we cannot exclude the possibility that the capsule remains a site of low-grade inflammation that would escape the detection by FDG-PET.

### Limitations

This study has some limitations. First, our measurement of the extent of neocortical or cerebellar hypometabolism was an underestimation because we did not consider the convoluted surface of the neocortex or the foliar structure of the cerebellar hemispheres. Therefore, the actual extent of neocortical or cerebellar hypometabolism was even greater than that measured with our procedure. Second, small abscesses (<0.5 cm^3^) did not cause detectable reduction of FDG uptake. We do not know whether this finding reflected the absence of damage to brain tissue or the inability of FDG-PET/CT to identify small areas of hypometabolism. PET is prone to “partial volume effects,” meaning that small areas of hypometabolism may escape detection because of the limited spatial resolution.

## CONCLUSION

A brain abscess leads to long-lasting focal hypometabolism, indicating tissue damage. The larger the abscess grows, the greater becomes the extent of the subsequent hypometabolism. This conclusion points to the importance of rapid neurosurgical intervention in brain abscess to minimize tissue damage and ensuing focal cerebral hypoactivity.

## Supplementary Material

SUPPLEMENTARY MATERIAL

## SUPPLEMENTAL DIGITAL CONTENT

**Supplemental Digital Content 1. Figure 1.** Flow chart of patient recruitment. Fifty patients were invited to take part in the study. Seven declined the invitation, 3 were excluded because their FDG-PET/CT findings could be attributed to conditions other than brain abscess: meningioma surgery (n = 2) and foreign body (n = 1). Thus 40 patients took part in the study and underwent MRI and FDG-PET/CT. Findings in these 40 patients underlie the correlation between abscess dimension and extent of cortical hypometabolism. For 9 patients, follow-up MRIs performed ≥1 year after brain abscess surgery were available for the evaluation of long-term structural effects of brain abscesses. **Supplemental Digital Content 2. Figure 2.** Images of 9 patients with brain abscess and subsequent focal tissue loss and gliosis. Forty patients with brain abscess who had been treated with neurosurgical drainage of pus plus long-term antibiotics. For 9 patients, follow-up MRIs more than 1 year after neurosurgery were available. **Supplemental Digital Content 3. Figure 3.** Images of 9 patients with brain abscess and subsequent focal hypometabolism that coincides with the peri-abscess edema that occurred in the acute phase of the disease. Forty patients with brain abscess who had been treated with neurosurgical drainage of pus plus long-term antibiotics underwent FDG-PET/CT days to years after neurosurgery. Focal hypometabolism was evident in 38 patients. In 9 patients, hypometabolism coincided with the peri-abscess edema although in most, the edema had resolved at the time of FDG-PET/CT investigation. In the next pages, the 9 patients are presented with those who underwent FDG-PET/CT closest to neurosurgery first.
