# Supplementary material for: Brain Abscess Causes Brain Damage With Long-Lasting Focal Cerebral Hypoactivity that Correlates With Abscess Size: A Cross-Sectional 18F-Fluoro-Deoxyglucose Positron Emission Tomography Study
Source: Neurosurgery. 2024 Nov 11;97(1):138–47. doi: 10.1227/neu.0000000000003268 (PMC12144652; doi:10.1227/neu.0000000000003268)
Supplement: SUPPLEMENTARY MATERIAL [file neu-97-138-s001.pptx]

## Slide 1
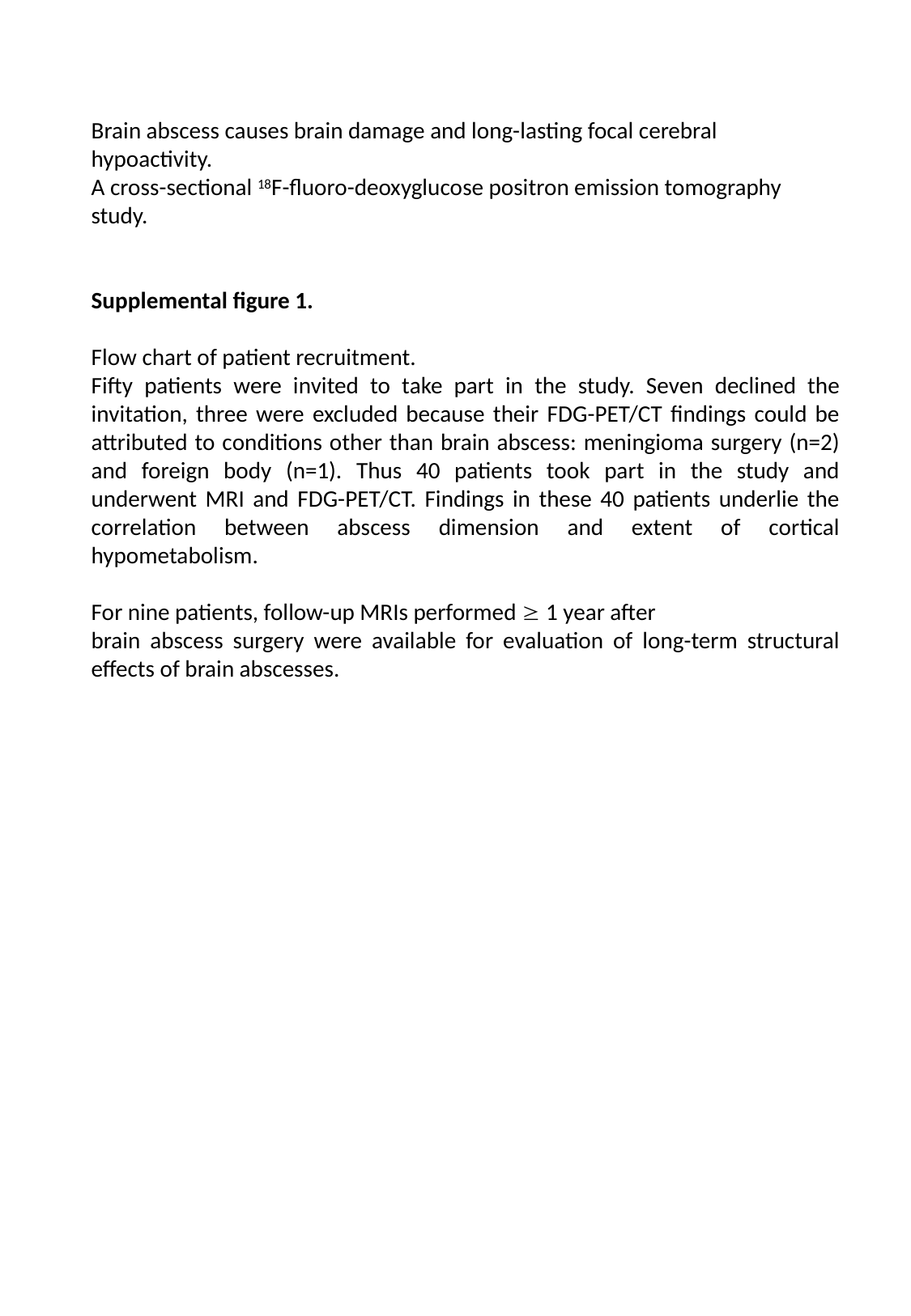

Brain abscess causes brain damage and long-lasting focal cerebral hypoactivity.
A cross-sectional 18F-fluoro-deoxyglucose positron emission tomography study.
Supplemental figure 1.
Flow chart of patient recruitment.
Fifty patients were invited to take part in the study. Seven declined the invitation, three were excluded because their FDG-PET/CT findings could be attributed to conditions other than brain abscess: meningioma surgery (n=2) and foreign body (n=1). Thus 40 patients took part in the study and underwent MRI and FDG-PET/CT. Findings in these 40 patients underlie the correlation between abscess dimension and extent of cortical hypometabolism.
For nine patients, follow-up MRIs performed ≥ 1 year after
brain abscess surgery were available for evaluation of long-term structural effects of brain abscesses.

## Slide 2
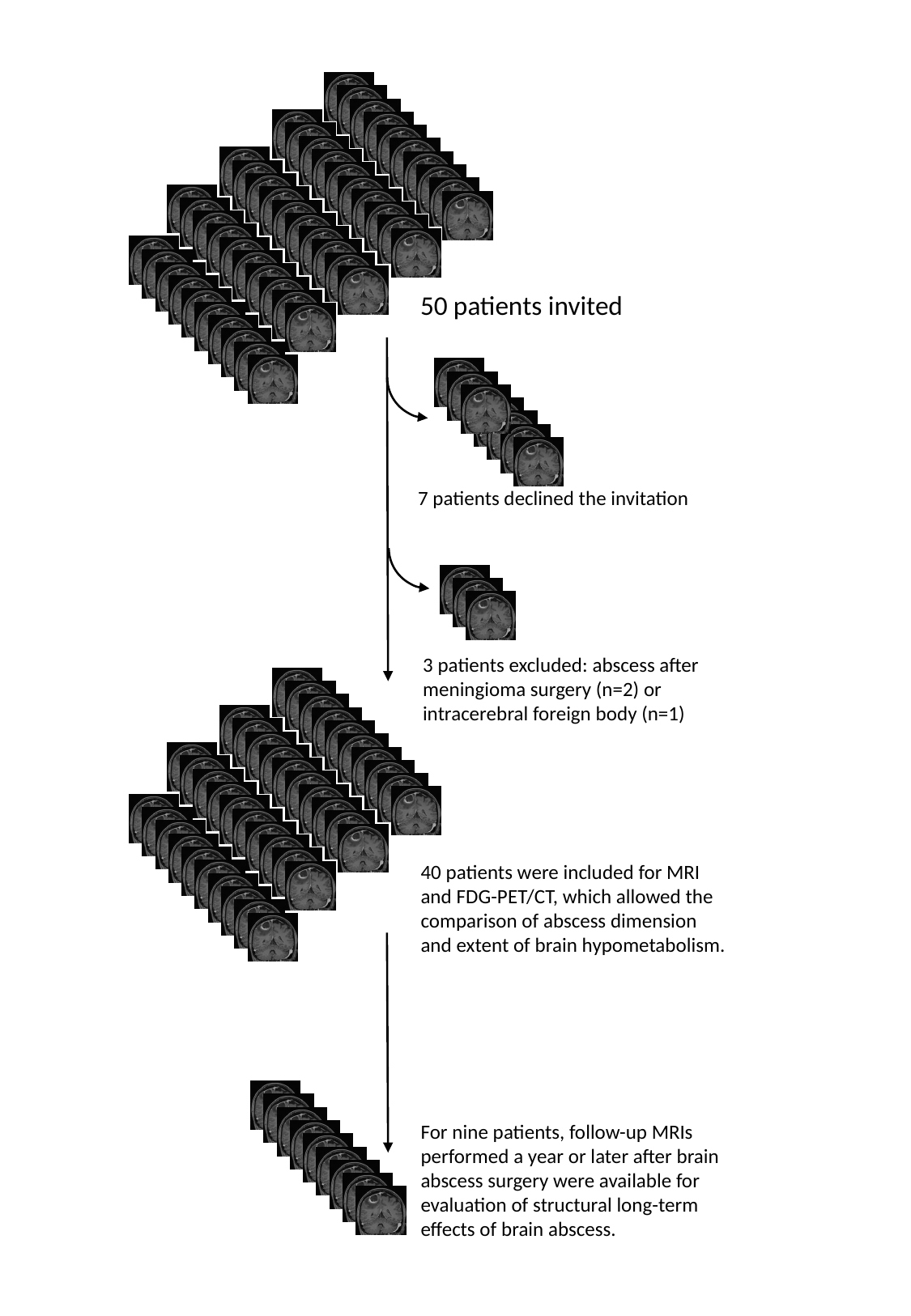

50 patients invited
7 patients declined the invitation
3 patients excluded: abscess after
meningioma surgery (n=2) or
intracerebral foreign body (n=1)
40 patients were included for MRI and FDG-PET/CT, which allowed the comparison of abscess dimension and extent of brain hypometabolism.
For nine patients, follow-up MRIs performed a year or later after brain abscess surgery were available for evaluation of structural long-term effects of brain abscess.
