# Supplementary material for: Brain Abscess Causes Brain Damage With Long-Lasting Focal Cerebral Hypoactivity that Correlates With Abscess Size: A Cross-Sectional 18F-Fluoro-Deoxyglucose Positron Emission Tomography Study
Source: Neurosurgery. 2024 Nov 11;97(1):138–47. doi: 10.1227/neu.0000000000003268 (PMC12144652; doi:10.1227/neu.0000000000003268)
Supplement: SUPPLEMENTARY MATERIAL [file neu-97-138-s002.pptx]

## Slide 1
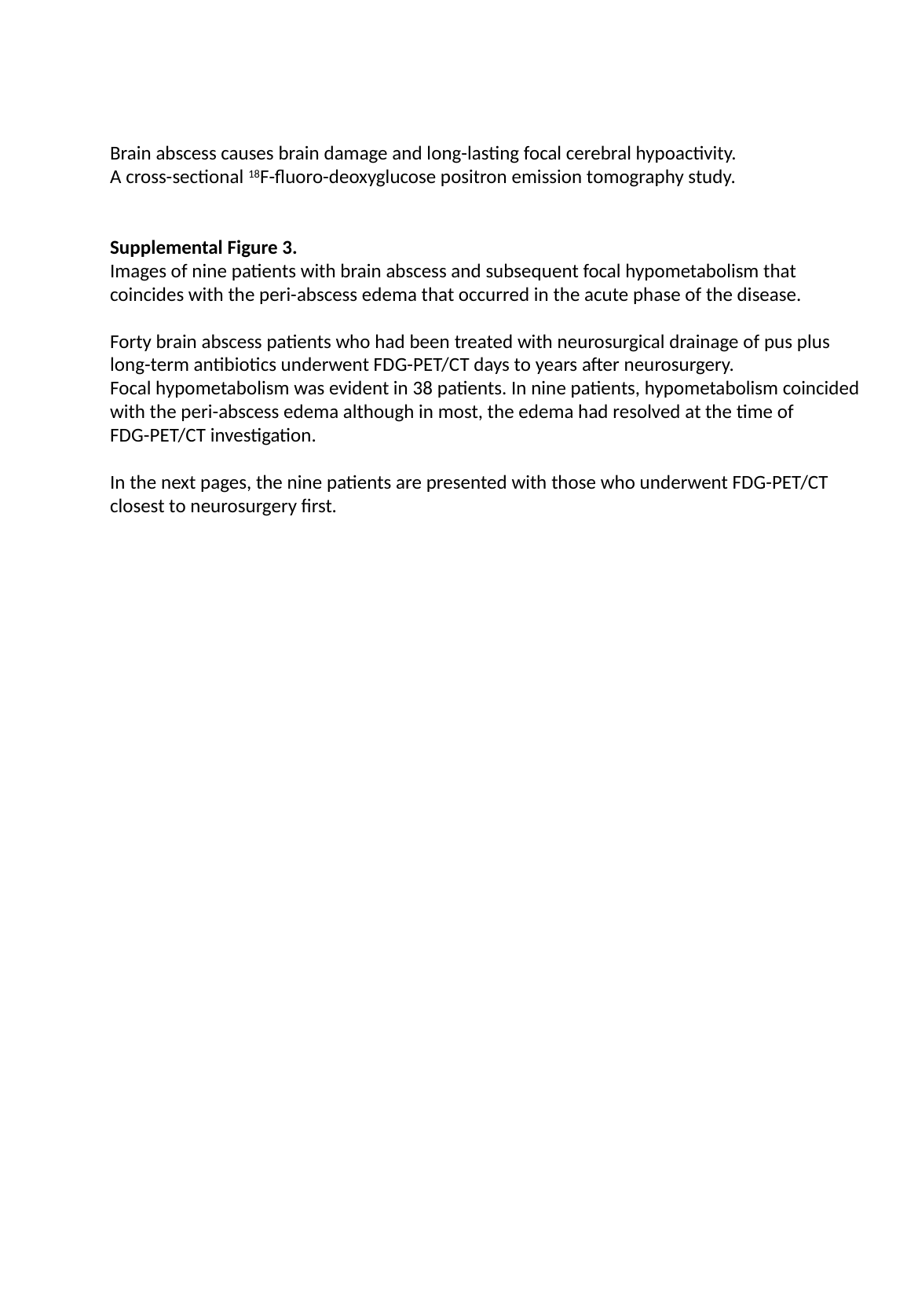

Brain abscess causes brain damage and long-lasting focal cerebral hypoactivity.
A cross-sectional 18F-fluoro-deoxyglucose positron emission tomography study.
Supplemental Figure 3.
Images of nine patients with brain abscess and subsequent focal hypometabolism that
coincides with the peri-abscess edema that occurred in the acute phase of the disease.
Forty brain abscess patients who had been treated with neurosurgical drainage of pus plus
long-term antibiotics underwent FDG-PET/CT days to years after neurosurgery.
Focal hypometabolism was evident in 38 patients. In nine patients, hypometabolism coincided
with the peri-abscess edema although in most, the edema had resolved at the time of
FDG-PET/CT investigation.
In the next pages, the nine patients are presented with those who underwent FDG-PET/CT
closest to neurosurgery first.

## Slide 2
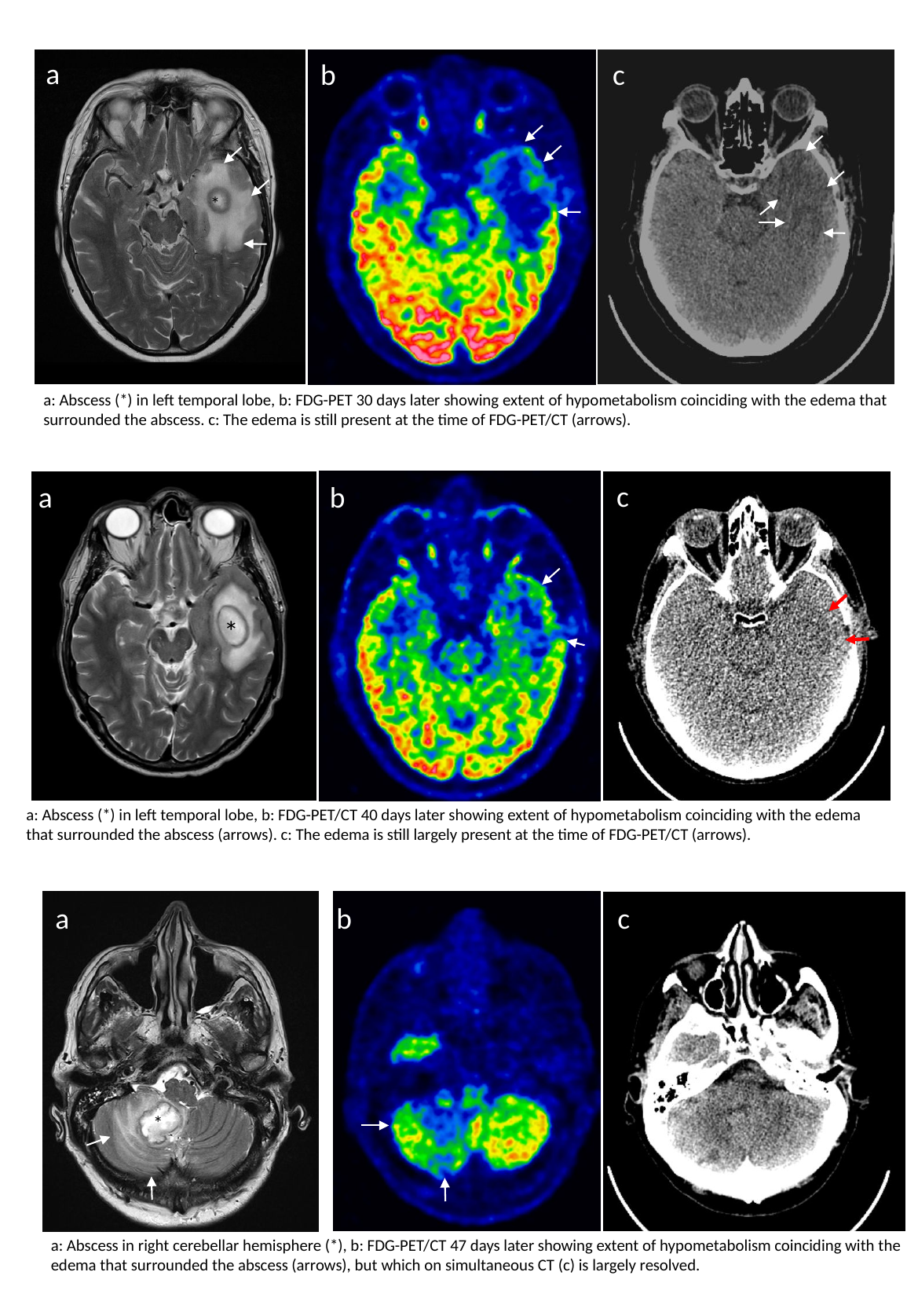

a
b
a: Abscess (*) in left temporal lobe, b: FDG-PET 30 days later showing extent of hypometabolism coinciding with the edema that surrounded the abscess. c: The edema is still present at the time of FDG-PET/CT (arrows).
*
c
c
a
b
a: Abscess (*) in left temporal lobe, b: FDG-PET/CT 40 days later showing extent of hypometabolism coinciding with the edema that surrounded the abscess (arrows). c: The edema is still largely present at the time of FDG-PET/CT (arrows).
*
*
a
b
a: Abscess in right cerebellar hemisphere (*), b: FDG-PET/CT 47 days later showing extent of hypometabolism coinciding with the edema that surrounded the abscess (arrows), but which on simultaneous CT (c) is largely resolved.
c
*

## Slide 3
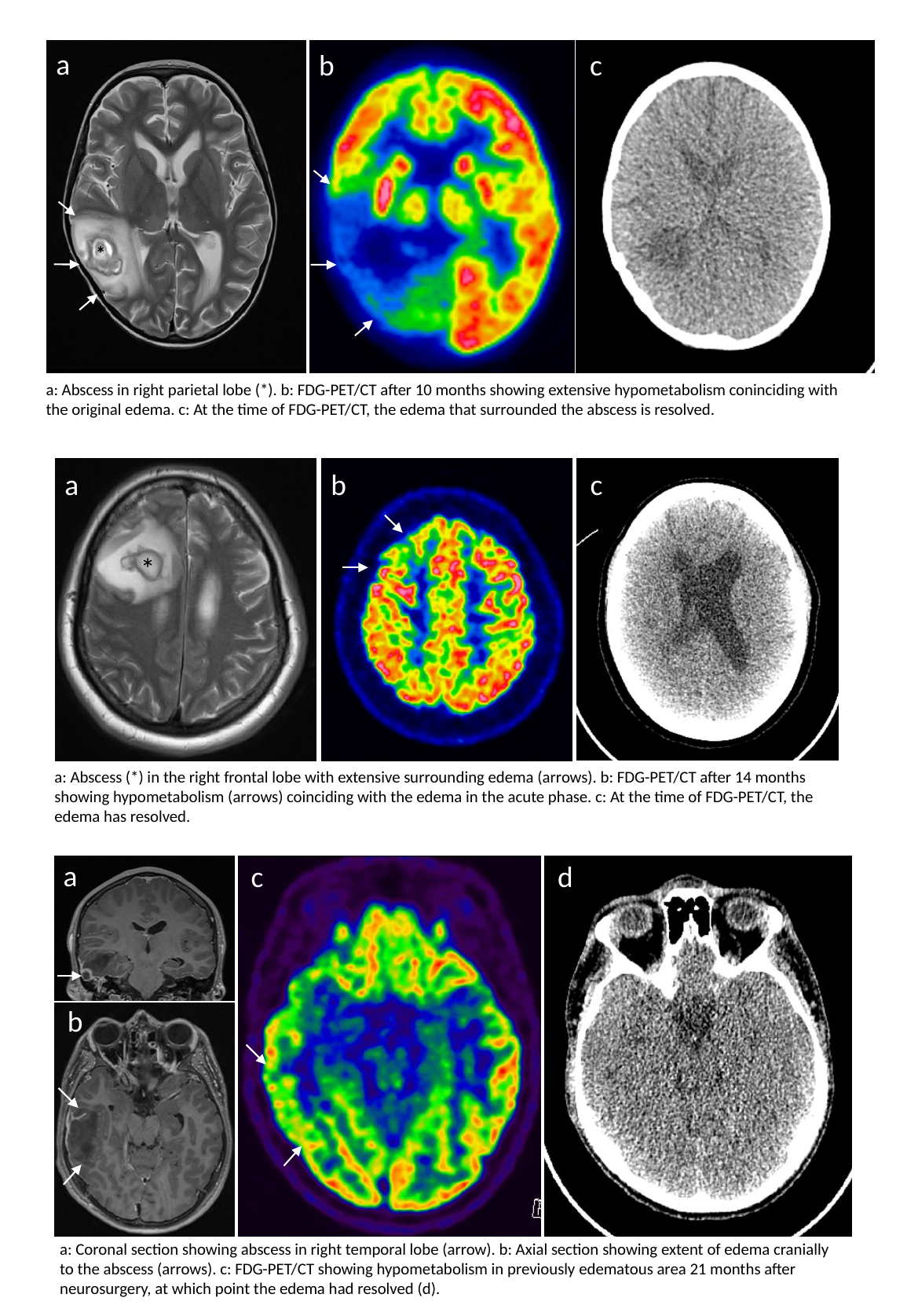

a
b
c
a: Abscess in right parietal lobe (*). b: FDG-PET/CT after 10 months showing extensive hypometabolism coninciding with the original edema. c: At the time of FDG-PET/CT, the edema that surrounded the abscess is resolved.
a
*
c
a
b
a: Abscess (*) in the right frontal lobe with extensive surrounding edema (arrows). b: FDG-PET/CT after 14 months showing hypometabolism (arrows) coinciding with the edema in the acute phase. c: At the time of FDG-PET/CT, the edema has resolved.
*
*
a
c
d
b
a: Coronal section showing abscess in right temporal lobe (arrow). b: Axial section showing extent of edema cranially to the abscess (arrows). c: FDG-PET/CT showing hypometabolism in previously edematous area 21 months after neurosurgery, at which point the edema had resolved (d).

## Slide 4
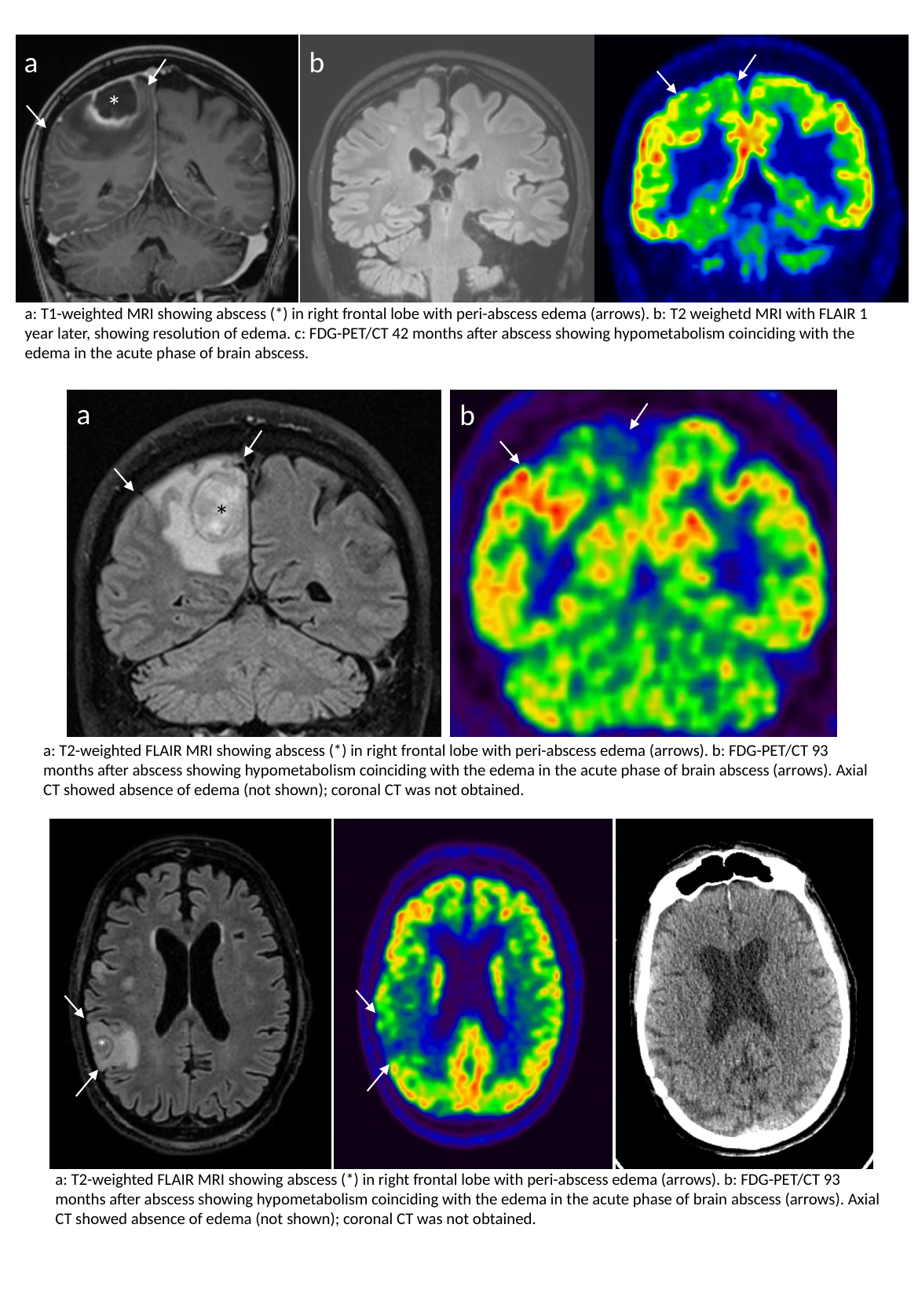

a
b
c
a: T1-weighted MRI showing abscess (*) in right frontal lobe with peri-abscess edema (arrows). b: T2 weighetd MRI with FLAIR 1 year later, showing resolution of edema. c: FDG-PET/CT 42 months after abscess showing hypometabolism coinciding with the edema in the acute phase of brain abscess.
*
a
a: T2-weighted FLAIR MRI showing abscess (*) in right frontal lobe with peri-abscess edema (arrows). b: FDG-PET/CT 93 months after abscess showing hypometabolism coinciding with the edema in the acute phase of brain abscess (arrows). Axial CT showed absence of edema (not shown); coronal CT was not obtained.
b
*
*
a: T2-weighted FLAIR MRI showing abscess (*) in right frontal lobe with peri-abscess edema (arrows). b: FDG-PET/CT 93 months after abscess showing hypometabolism coinciding with the edema in the acute phase of brain abscess (arrows). Axial CT showed absence of edema (not shown); coronal CT was not obtained.
